# Association between delayed thromboprophylaxis and the incidence of deep vein thrombosis in patients with pelvic trauma: a retrospective cohort adjusted for severity at a level I trauma center

**DOI:** 10.1590/1677-5449.202600402

**Published:** 2026-09-07

**Authors:** Jihad Manaf El Sayed, Guilherme Marum Olmedo, Adilia Maria Pires Sciarra, Lilian Castiglioni, Paulo César Espada

**Affiliations:** 1 Faculdade de Medicina de São José do Rio Preto – FAMERP, São José do Rio Preto, SP, Brasil.

**Keywords:** pelvis, wounds and injuries, venous thrombosis, disease prevention, anticoagulants

## Abstract

**Background:**

Patients with pelvic trauma are at high risk for deep vein thrombosis (DVT), but early initiation of thromboprophylaxis may increase bleeding risk in hemodynamically unstable individuals. The optimal timing for initiation of anticoagulation remains controversial.

**Objectives:**

To evaluate whether delayed initiation of pharmacologic prophylaxis is associated with a higher incidence of DVT in patients with pelvic trauma, after adjustment for age and activation of the massive transfusion protocol.

**Methods:**

This retrospective observational cohort study was conducted at a Level I trauma center from January 2022 to December 2023. Adult patients with computed tomography–confirmed pelvic trauma who underwent Doppler ultrasonography during hospitalization were included. The primary exposure was initiation of pharmacologic prophylaxis within 48 hours (early) vs. after 48 hours (delayed). The primary outcome was occurrence of DVT during hospitalization. Multivariable logistic regression was conducted, adjusting for age and activation of the massive transfusion protocol.

**Results:**

A total of 62 patients were analyzed, with a mean age of 47.71 ± 19.91 years and predominance of male sex (66.1%). The overall incidence of DVT was 32.3% (n=20). In the bivariate analysis, DVT incidence was 35.3% in the delayed group and 28.6% in the early group (p = 0.598). In the multivariable analysis, early initiation was not independently associated with the occurrence of DVT (OR 0.65; 95% CI 0.21–2.00; p = 0.456).

**Conclusions:**

Delayed initiation of thromboprophylaxis was not independently associated with a higher incidence of DVT after adjustment for age and activation of the massive transfusion protocol.

## INTRODUCTION

Severe pelvic trauma is a highly complex clinical condition with considerable systemic impact. It is associated both with early hemorrhagic complications and potentially fatal thromboembolic events. These injuries are generally caused by high energy mechanisms such as automobile collisions and falls from great height and cause significant early mortality, predominantly due to massive hemorrhage and hypovolemic shock.^1^ The rich arterial and venous vascularization of the pelvis, combined with the proximity of visceral structures and large venous plexuses, make large scale blood loss likely, often demanding intervention for damage control.

However, from a vascular perspective, pelvic trauma cannot be understood merely as an acute hemorrhagic event, because it is also a potent cause of prothrombotic states. Patients with fractures involving the pelvic ring or acetabula are subject to a greatly increased risk of venous thromboembolism (VTE), including deep venous thrombosis (DVT) and pulmonary embolism.^2^ The pathophysiology of these processes is multifactorial and reflects Virchow’s classic triad: endothelial damage caused by the trauma and by surgical procedures; stasis caused by prolonged immobilization and extrinsic vein compression; and a hypercoagulable state caused by the systemic inflammatory response and activation of the hemostatic cascade in the context of polytrauma.^3^ The combination of these mechanisms creates a persistent thrombogenic environment, which can last for weeks after the initial insult.

International guidelines recommend early introduction of pharmacological prophylaxis for VTE in trauma patients, as soon as the risk of hemorrhage has been adequately controlled.^4^ However, specifically in severe pelvic trauma scenarios, this decision often involves a relevant clinical dilemma. In patients with active bleeding or hemodynamic instability or those needing repeated surgical interventions, early anticoagulation can theoretically exacerbate blood loss or compromise strategies for bleeding control.^5^ Therefore, the balance between thrombotic risk and hemorrhagic risk plays a central role in decision making, especially at high complexity trauma centers.

In clinical practice, it is observed that patients with greater physiological severity tend to be given pharmacological thromboprophylaxis later, after hemodynamic stabilization and definitive or temporary control of bleeding. While this conduct is understandable from a hemostatic perspective, concerns remain that delayed anticoagulation could amplify the risk of thrombotic events, particularly in patients who are already exposed to intense inflammatory and transfusional stimuli. Previous observational studies have reported mixed results, which are often limited by non-specific trauma patient samples and by a lack of appropriate adjustment for objective markers of clinical severity.^6^

From the point of view of vascular surgery and of hospital vascular medicine, it is essential to determine whether the time at which thromboprophylaxis is started has an independent impact on the incidence of DVT in patients with pelvic trauma, or if thromboembolic risk is predominantly determined by the magnitude of the systemic physiological response to the trauma. Identification of independent determinants of DVT in this scenario could contribute to development of more individualized prophylaxis strategies, with better balance between hemorrhagic safety and antithrombotic protection.

Against this background, the objective of this study was to assess whether, in a specific population of patients with pelvic trauma seen at a level I trauma center, delayed introduction of pharmacological prophylaxis would be associated with a higher incidence of DVT is than early introduction, after adjustment for clinical severity variables. The hypothesis tested was that, after consideration of objective markers of hemorrhagic severity and instability, delayed introduction of anticoagulation would not be independently associated with increased DVT incidence while in hospital.

## METHODS

This was an observational, retrospective cohort study conducted at a high complexity tertiary hospital, classified as a level I trauma center, the Hospital de Base de São José do Rio Preto, in São Paulo state, Brazil. The enrollment period was from January 2022 to December 2023. The study was approved by the institution’s Research Ethics Committee (CAAE submission number: 67737823.0.0000.5415, opinion number 6.050.468).

Patients were identified by means of a systematic search of the trauma service’s database, using electronic hospital records, physical medical charts, and a diagnostic coding system. First, all adult patients admitted for trauma during the study period were identified. Then a specific filter was applied to identify those with a diagnosis of pelvic injury confirmed by computed tomography conducted at hospital admission. The definition of pelvic trauma included fractures of the pelvic ring, acetabular fractures, and associated complex injuries, irrespective of the need for surgical treatment.

After this initial screening, pre-established eligibility criteria were applied. Patients aged 18 years or over, with tomographic confirmation of pelvic injury, and a hospital stay long enough to assess the thrombotic outcome were included. Patients were excluded if they died in the first 48 hours after admission, considering that this period does not allow enough clinical time for systematic implementation of pharmacological prophylaxis and outcome monitoring. Patients were also excluded if they had absolute contraindications to anticoagulation for the entire hospital stay, such as non-stabilized intracranial bleeding or severe persistent coagulopathy, as were patients who did not have Doppler ultrasonography of the lower limbs while in hospital, making objective assessment of the primary outcome impossible. After sequential application of these criteria, a final sample was selected comprising 62 patients eligible for analysis. The process followed the STROBE recommendations for transparent description of participant flows.

Data were collected by detailed review of electronic and physical patient records, extracting demographic variables, trauma mechanism, clinical parameters at admission, need for surgical interventions, use of blood products, length of hospital stay, type and time of initiation of pharmacological prophylaxis, and occurrence of DVT. Indicators of physiological severity were recorded, including Shock Index and activation of the massive transfusion protocol, in addition to presence of clinical signs of shock at admission.

The primary exposure was time elapsed until initiation of pharmacological prophylaxis for DVT, categorized into two groups: early initiation, defined as administration of the first dose of anticoagulant within 48 hours of hospital admission; and delayed initiation, defined as first administration more than 48 hours after admission. The 48-hour cutoff point was set on the basis of consolidated institutional clinical practices and evidence reported in the specialist literature. The decision on when to start anticoagulation was taken on a case-by-case basis by the care team, considering hemodynamic stability, need for blood transfusion, presence of active bleeding, findings of imaging exams, need for surgical or endovascular interventions, and overall hemorrhagic risk assessment.

The primary outcome was occurrence of DVT diagnosed by Doppler ultrasonography of the lower limbs while in hospital. DVT was defined as present when thrombosis was identified in the deep vein system, irrespective of clinical manifestations. Doppler ultrasonography of the lower limbs was employed in accordance with the institution’s protocol; conducted systematically for all patients in the intensive care unit (ICU) from their fifth day in hospital or in the event of clinical suspicion of DVT. For patients in general wards, ultrasonography was requested on the basis of the trauma team’s standardized care criteria, considering thromboembolic risk factors and clinical progress. All of the patients selected for the sample underwent at least one Doppler ultrasonography scan of the lower limbs while in hospital, irrespective of presence of clinical symptoms. Thus, the thrombotic outcome was assessed in accordance with a preestablished institutional care protocol, ensuring that all patients underwent at least one objective DVT assessment while in hospital.

While the retrospective design limits causal inferences, the adjusted analysis was intended to mitigate potential clinically relevant confounding factors, contributing to a more robust interpretation of the findings.

Statistical analysis was conducted in sequential steps. First, a descriptive analysis of the variables was conducted, calculating measures of central tendency and dispersion for quantitative variables and absolute and relative frequencies for categorical variables. Categorical variables were compared using the chi-square test or Fisher’s exact test, when appropriate. The Kolmogorov-Smirnov test was used to check the normality of quantitative data. In view of the nonparametric distribution of the majority of continuous variables, the Mann-Whitney test was used to compare independent groups. Correlation analyses were performed using Spearman coefficients, classifying magnitude according to predefined values. The significance level adopted was p ≤ 0.05.

The association between time anticoagulation was started and occurrence of DVT was initially tested using univariate analysis. Then, a multivariable logistic regression was estimated with occurrence of DVT (yes/no) as dependent variable and anticoagulation initiation time (early vs. delayed) as main independent variable. Considering the limited number of events observed, the final multivariable model was restricted to three variables, selected on the basis of clinical plausibility and relevance as potential confounding factors, in order to avoid overadjustment and preserve the stability of estimates. Thus, in addition to the primary exposure variable, age (continuous variable) and activation of the massive transfusion protocol (as a marker of physiological severity) were included in the model. Estimates were expressed as odds ratios with their respective 95% CI. There were no missing values for the main variables analyzed.

Since this was a retrospective study based on a convenience sample, all eligible patients identified during the pre-established period were included after application of the inclusion and exclusion criteria. No sample size calculation was performed prior to the study to determine the number of participants, since the sample size was determined by the availability of cases in the institution’s database during the time period analyzed. Nonetheless, an estimate of the ideal sample size for comparison of two independent proportions was calculated for the purposes of interpretation. This calculation used the proportions of DVT observed in the early and delayed pharmacological thromboprophylaxis initiation groups, for a 5% significance level and statistical power of 80%, parameters conventionally used in sample calculations for clinical studies. This analysis was used to contextualize the precision of estimates and the study’s capacity to detect differences between the two groups.

## RESULTS

A total of 62 patients with a diagnosis of blunt pelvic trauma were included. Mean age was 47.71 ± 19.91 years, demonstrating the wide age range within this cohort. A majority of the sample was male (66.1%) and the trauma mechanism was classified as high kinetic energy in 80.6% of cases, characterizing a population exposed to a high degree of energy transfer. During pre-hospital care, 21% of the patients showed clinical signs of shock. Volume resuscitation was performed with crystalloids in 38.7% of the sample, breaking down as 25.8% with volumes ≤ 1000 mL and 12.9% with volumes exceeding 1000 mL. Tranexamic acid was administered in the pre-hospital setting in 17.7% of cases.

At hospital admission, 8.1% of the patients had clinical signs of hypovolemia and 11.3% had evident active bleeding. The massive transfusion protocol was activated in 8.1% of the sample. Mean Shock Index was 0.82 ± 0.37 with a median of 0.76, revealing the considerable variation in initial hemodynamic response. The ABC score distribution was predominantly low, with 74.2% of the patients scoring 0, 17.7% scoring 1, 6.5% scoring 2, and 1.6% scoring 3.

Associated fractures were identified in other anatomic areas in 59.7% of the patients, indicating a high frequency of polytrauma. Damage control surgery was necessary in 14.5% of the sample. Surgical correction of a pelvic fracture was performed in 58.1% of cases, while 40.3% underwent repair of other associated fractures. The mean ICU stay was 12.98 ± 14.46 days, with a median of 7 days, while the mean time spent in a ward was 15.08 ± 17.10 days with a median of 9 days, revealing highly variable clinical progress while in hospital (Table 1).

**Table 1:** Baseline demographic and clinical characteristics (n = 62).

Variable	Value
Age (years)	47.71 ± 19.91
Male sex	66.1%
High energy trauma	80.6%
Pre-hospital shock	21%
Resuscitation with crystalloids	38.7%
≤ 1000 mL	25.8%
> 1000 mL	12.9%
Tranexamic acid before admission	17.7%
Hypovolemia at admission	8.1%
Active bleeding evident	11.3%
Massive transfusion protocol	8.1%
Shock Index (mean ± SD)	0.82 ± 0.37
Shock Index (median)	0.76
ABC score 0	74.2%
ABC score 1	17.7%
ABC score 2	6.5%
ABC score 3	1.6%
Additional fractures	59.7%
Damage control surgery	14.5%
Pelvic fracture surgery	58.1%
Surgery for other fractures	40.3%
ICU (days, mean ± SD)	12.98 ± 14.46
ICU (median)	7
Ward (days, mean ± SD)	15.08 ± 17.10
Ward (median)	9

ICU = intensive care unit.

All patients were given pharmacological prophylaxis for VTE while in hospital. Enoxaparin was used in 54.8% of cases, while 45.2% were given unfractionated heparin. Prophylaxis was initiated in under 48 hours after admission in 45.2% of the patients, whereas 54.8% were started on prophylaxis after more than 48 hours. The mean time until initiation of anticoagulation was 3.44 ± 2.15 days. Table 2 shows a detailed comparison of baseline variables between groups with early (≤ 48 hours) and delayed (> 48 hours) initiation of pharmacological prophylaxis. No statistically significant differences between the groups were observed for age, Shock Index, presence of shock at admission, or need for damage control surgery, although the delayed initiation group had a higher proportion of patients for whom the massive transfusion protocol was activated. This descriptive analysis was conducted to assess possible confounding by indication.

**Table 2:** Comparison between early (≤ 48h) and delayed (> 48h) initiation.

Variable	≤ 48h (n = 28)	> 48h (n = 34)	p
Age (mean ± SD)	46.36 ± 18.52	48.85 ± 21.10	0.642
Shock Index (mean ± SD)	0.79 ± 0.31	0.85 ± 0.41	0.588
Shock at admission (%)	7.1%	8.8%	0.812
Massive transfusion protocol (%)	3.6%	11.8%	0.284
Damage control surgery (%)	10.7%	17.6%	0.482

h = hours.

DVT was confirmed by Doppler ultrasonography in 32.3% of the patients (n = 20). The mean time until diagnosis was 19.17 ± 14.26 days after the trauma, with a median of 13.5 days and a range of 6-55 days. Fifty percent of the confirmed cases had edema at the time of diagnosis, 10% reported pain and 40% were asymptomatic, with DVT detected by ultrasound screening (Table 3).

**Table 3:** Anticoagulant Prophylaxis and Incidence of DVT.

Variable	Group	Value
Anticoagulant	Enoxaparin	54.8%
Anticoagulant	Unfractionated heparin	45.2%
Initiated prophylaxis	≤ 48 h	45.2%
Initiated prophylaxis	> 48 h	54.8%
Time until initiated (days)	Mean ± SD	3.44 ± 2.15
Incidence of DVT	Overall	32.3% (n = 20)
DVT – Initiated ≤ 48h	-	28.6% (8/28)
DVT – Initiated > 48h	-	35.3% (12/34)
Time until DVT (days)	Mean ± SD	19.17 ± 14.26
Time until DVT (days)	Median (range)	13.5 (6–55)
Symptomatic DVT	Edema	50%
Symptomatic DVT	Pain	10%
Asymptomatic DVT	-	40%

DVT = deep vein thrombosis; h = hours.

In the bivariate analysis, it was observed that 12 of the 34 patients (35.3%) who were started on anticoagulation after more than 48 hours developed DVT, while eight of the 28 patients (28.6%) who were started in less than 48 hours underwent the same event, equating to an absolute difference of 6.7 percentage points, which was not statistically significant (p = 0.598). The severity of pelvic trauma, estimated by the number of fractures identified, was not associated with occurrence of DVT (p = 0.7525).

A multivariable logistic regression was estimated with occurrence of DVT as the dependent variable. The final model was defined in advance on the basis of clinical plausibility and the limit imposed by the number of events observed, in order to reduce the risk of overadjustment and preserve the stability of estimates. Therefore, three variables were included in the adjusted model: early initiation of anticoagulation (≤ 48 hours), as main independent variable of interest; age, considered a continuous variable with a potential biological influence on thrombotic risk; and activation of the massive transfusion protocol, used as an objective marker of physiological severity and initial hemorrhagic instability.

In the adjusted multivariable model, early anticoagulation initiation had an odds ratio of 0.65 (95% CI, 0.21–2.00; p = 0.456). The wide CI observed reflects the imprecision of the estimates, which is compatible with the limited number of events in the cohort. Age had an odds ratio of 1.01 for each additional year (95% CI, 0.98–1.04; p = 0.452). Activation of the massive transfusion protocol had an odds ratio of 16.09 (95% CI, 0.89–292.13; p = 0.061), also with a wide CI, so its magnitude should be interpreted with care (Table 4).

**Table 4:** Multivariate logistic regression for occurrence of DVT.

Variable	Adjusted OR	95%CI	p
Early anticoagulation (≤ 48h)	0.65	0.21–2.00	0.456
Massive transfusion protocol	16.09	0.89–292.13	0.061
Age (per additional year)	1.01	0.98–1.04	0.452

DVT = deep vein thrombosis; h = hours; OR = odds ratio.

The point estimate of less than one for early anticoagulation initiation remained after adjustment for age and hemorrhagic severity, with no evidence of an independent association with DVT occurrence in the analyzed cohort. Activation of the massive transfusion protocol had the greatest magnitude of association in the model, underscoring the relevance of initial physiological severity as a central component in stratification of thrombotic risk in patients with pelvic trauma. These findings suggest that the systemic profile of hemorrhagic instability may exert a more consistent influence over the outcome than the specific interval of time elapsed before introduction of pharmacological prophylaxis.

## DISCUSSION

DVT in the context of pelvic trauma remains a very important clinical challenge, even at centers set up for trauma care and with systematic use of pharmacological prophylaxis. The literature shows that fractures involving the pelvic ring and acetabula are among the orthopedic injuries with greatest risk of VTE, particularly when in conjunction with polytrauma, need for intensive care, and multiple surgical interventions.^1,7,8^ As such, the incidence observed in the present cohort should not be interpreted as an isolated event, but as an expected manifestation of a highly thrombogenic biological phenotype.

The central finding of this study — the absence of an independent association between delayed initiation of thromboprophylaxis and increased occurrence of DVT after adjustment for age and activation of the massive transfusion protocol — merits careful analysis. European and international guidelines recommend early initiation of pharmacological prophylaxis as soon as the risk of hemorrhage is controlled,^4^ but recognize that patients with severe pelvic trauma often have hemodynamic instability and need transfusions, both of which are factors that can delay prudent anticoagulation.^9,10^ Recent prospective studies suggest that early initiation of low molecular weight heparin is safe in stabilized pelvic fractures,^11,12^ but these studies generally include populations with rigorous criteria for stabilization and lower proportions of patients who undergo massive transfusion.

The limited number of events observed restricts the statistical power to detect modest differences between groups. The wide CI for the primary exposure variable suggests a possibility of type II error, so it is not possible to entirely rule out the existence of an association with small or moderate magnitude between time to initiation of prophylaxis and occurrence of DVT.

While performing a sample size calculation in advance was not obligatory for the retrospective design employed, based on the inclusion of all eligible patients during the analyzed period, the supplementary estimate of an ideal sample size contributes to contextualize the precision of the findings. Considering the observed proportions of DVT in the early and delayed pharmacological thromboprophylaxis initiation groups of 28.6% and 35.3%, respectively, with an absolute difference of 6.7 percentage points, a 5% significance level, and 80% statistical power, a sample of approximately 759 patients per group, totaling around 1,518 patients in a 1:1 distribution, would be needed to detect a difference of this magnitude between the groups. In view of this, the absence of statistical significance observed in this cohort should be interpreted with care, since it could reflect insufficient statistical power to identify discrete differences between the analyzed strategies. These data underscore that the results of the study should be understood as exploratory and as hypothesis generators, rather than as a definitive demonstration of the equivalence of early and delayed initiation of pharmacological thromboprophylaxis.

Adjustment for age and activation of the massive transfusion protocol, as an objective marker of physiological severity, was intended to reduce the confounding by indication bias that is often present in retrospective studies. More severe patients tend to receive anticoagulation later and, simultaneously, have greater intrinsic thrombotic risk. In this context, the results indicate that the initial systemic severity may have a more consistent influence on risk of DVT than the specific interval of time elapsed before introduction of pharmacological prophylaxis.

One particularly relevant aspect was the magnitude of the associations observed for activation of the massive transfusion protocol. The literature shows that patients who undergo massive transfusion have profound changes to hemostasis, including initial coagulopathy followed by a reactive hypercoagulable state.^4,13,14^ The exacerbated systemic inflammatory response, diffuse endothelial injury, and platelet activation contribute to a persistent prothrombotic environment.^3^ Therefore, the results highlight that the thromboembolic risk associated with pelvic trauma is multifactorial and powerfully influenced by the patient’s overall physiological condition.

The need for damage control surgery merits reflection in the clinical context of severe pelvic trauma. The abbreviated procedures often conducted in unstable patients prioritize hemorrhagic control and physiological stabilization.^15,16^ These patients accumulate additional prothrombotic factors, such as prolonged immobilization, multiple reoperations, and long stays in the ICU. While this variable was not included in the final multivariable model, it constitutes a clinical marker of severity that may contribute to greater thromboembolic vulnerability in scenarios of accentuated physiological instability.

Another relevant point is related to the time until diagnosis of DVT, which predominantly occurred in later phases of the hospital stay. This finding is in agreement with studies demonstrating that thrombotic risk remains throughout the hospital stay, even after initial stabilization.^7,8,17^ Post-traumatic hypercoagulability can last for weeks, influenced by a continuous inflammatory response and repeated surgical stimuli.^3^ As such, prevention of VTE after pelvic trauma must be understood as a longitudinal strategy, and not only as an isolated early intervention.

The proportion of asymptomatic events detected by ultrasonography underscores the importance of diagnostic screening. Studies using serial Doppler scans demonstrate a substantial incidence of subclinical DVT in pelvic and acetabular fractures.^7,8^ The absence of a universal screening protocol can introduce a detection bias, influencing the observed rate and making comparisons between different centers difficult. However, this scenario reflects the true clinical practice at many centers, which confers external applicability on the findings.

With regard to the type of anticoagulant used, meta-analyses indicate that low molecular weight heparin may offer a discrete advantage for prevention of VTE in trauma patients.^18^ However, institutional factors, dosage variations, and individual characteristics can attenuate differences between agents. The absence of an independent association between type of heparin and occurrence of DVT in the present analysis suggests that, in the studied setting, the severity of trauma and the patient’s physiological status play a more determinant role than the specific drug chosen.

Methodologically, the retrospective design imposes limitations, including the possibility of residual confounding by unmeasured variables, such as dynamic laboratory coagulation parameters, intensity of immobilization, or variations in early rehabilitation strategies. The limited number of events imposes a restriction on the number of variables that could be included in the final multivariable model, so residual confounding may remain. It was necessary to exclude patients who died in the first 48 hours to enable adequate assessment of the exposure, but this could have introduced survival bias.

In the domestic setting, the Brazilian Society of Angiology and Vascular Surgery recommendations on VTE^19^ stress the importance of individualized stratification of thrombotic and hemorrhagic risk in hospitalized patients, especially those who have undergone trauma, surgery, prolonged immobilization, or admission to an ICU. This perspective is particularly relevant in the context of severe pelvic trauma, in which powerful prothrombotic factors coexist, such as endothelial injury, systemic inflammatory response, venous stasis, polytrauma, need for surgical procedures, and prolonged periods bedridden, at the same time that the initial hemorrhagic risk may limit or delay safe introduction of pharmacological prophylaxis. Therefore, the findings of this study should be interpreted in the light of this clinical equilibrium: while pharmacological thromboprophylaxis should be initiated early whenever it is hemostatically safe to do so, the delay in physiologically unstable patients may reflect their greater underlying severity and not necessarily constitute an independent thrombotic risk factor. This interpretation is in line with the need, stressed in the Brazilian guidelines, to individualize VTE prevention to fit the clinical profile, risk of bleeding, and hemodynamic progress of each patient.

Despite these limitations, this study contributes by assessing a specific population with pelvic trauma at a level I trauma center, with adjustment for an objective marker of physiological severity. The findings suggest that thromboembolic risk is strongly influenced by the patient’s overall systemic condition.^4,10,20^ In this cohort, moderate delay to pharmacological prophylaxis, when clinically justified by hemodynamic instability or hemorrhagic risk, did not demonstrate an independent association with greater DVT incidence after adjustment for age and hemorrhagic severity. These results should be interpreted in the light of the methodological limitations described and underscore the need for case-by-case assessment of the balance between hemorrhagic risk and thrombotic risk in patients with severe pelvic trauma.

## CONCLUSIONS

In patients with blunt pelvic trauma treated at a level I trauma center, it was not possible to demonstrate an independent association between starting pharmacological thromboprophylaxis after more than 48 hours and higher incidence of DVT after adjustment for age and hemorrhagic severity. Considering the limited number of events and the width of the CI observed, studies with greater statistical power are needed to confirm these findings and better define the impact of the time elapsed before prophylaxis is initiated in this clinical scenario.

## DISPONIBILIDADE DE DADOS

Os dados que sustentam este estudo estão disponíveis mediante solicitação ao autor correspondente, JMES, pois seu compartilhamento está sujeito a restrições éticas e à preservação da confidencialidade dos participantes.
