# Supplementary material for: Association between delayed thromboprophylaxis and the incidence of deep vein thrombosis in patients with pelvic trauma: a retrospective cohort adjusted for severity at a level I trauma center
Source: J Vasc Bras. 2026 Sep 7;25:e20260040. doi: 10.1590/1677-5449.202600402 (PMC13671012; doi:10.1590/1677-5449.202600402)
Supplement: Translation

# Translation: Associação entre a tromboprofilaxia tardia e a incidência de trombose venosa profunda em pacientes com trauma pélvico: coorte retrospectiva ajustada para gravidade em um centro de trauma de nível I

**DOI:** 10.1590/1677-5449.202600401

**Authors:** Jihad Manaf El Sayed, Guilherme Marum Olmedo, Adilia Maria Pires Sciarra, Lilian Castiglioni, Paulo César Espada

**Affiliations:** 1 Faculdade de Medicina de São José do Rio Preto – FAMERP, São José do Rio Preto, SP, Brasil.

**Keywords:** pelve, trauma, trombose venosa profunda, profilaxia, anticoagulantes

## Abstract

**Contexto:**

Pacientes com trauma pélvico apresentam elevado risco de trombose venosa profunda, enquanto o início precoce da tromboprofilaxia pode aumentar o risco hemorrágico em indivíduos hemodinamicamente instáveis. O momento ideal para introdução da anticoagulação permanece controverso.

**Objetivos:**

Avaliar se o início tardio da profilaxia farmacológica está associado à ocorrência de trombose venosa profunda em pacientes com trauma pélvico após ajuste para idade e ativação do protocolo de transfusão maciça.

**Métodos:**

Estudo observacional de coorte retrospectiva, conduzido em um centro de trauma de nível I entre janeiro de 2022 e dezembro de 2023, incluiu pacientes adultos com trauma pélvico. A exposição principal foi início da tromboprofilaxia farmacológica em até 48 horas (precoce) *versus* após 48 horas (tardia). O desfecho primário foi ocorrência de trombose venosa profunda durante a internação. Realizou-se regressão logística multivariada ajustada para idade e ativação do protocolo de transfusão maciça.

**Resultados:**

Analisaram-se 62 pacientes, com idade média de 47,71±19,91 anos, e predominância do sexo masculino (66,1%). A incidência global de trombose venosa profunda foi de 32,3% (n = 20). Na análise bivariada, a incidência foi de 35,3% no grupo de início tardio e 28,6% no grupo de início precoce (p = 0,598). Na análise multivariada, o início precoce não se associou independentemente à ocorrência de trombose venosa profunda (*odds ratio* 0,65; intervalo de confiança de 95% 0,21-2,00; p = 0,456).

**Conclusões:**

O início tardio da tromboprofilaxia não apresentou associação independente com maior incidência de trombose venosa profunda após ajuste para idade e ativação do protocolo de transfusão maciça.

## INTRODUÇÃO

O trauma pélvico grave representa uma condição clínica de elevada complexidade e importante impacto sistêmico, associando-se tanto a complicações hemorrágicas precoces quanto a eventos tromboembólicos potencialmente fatais. Decorrente, em geral, de mecanismos de alta energia, como colisões automobilísticas e quedas de grande altura, esse tipo de lesão apresenta mortalidade inicial significativa, sobretudo em virtude de hemorragia maciça e choque hipovolêmico^1^. A rica vascularização arterial e venosa da pelve, aliada à proximidade de estruturas viscerais e grandes plexos venosos, favorece perdas sanguíneas expressivas e frequentemente demanda intervenções para o controle de danos.

Sob a perspectiva vascular, entretanto, o trauma pélvico não deve ser compreendido apenas como um evento hemorrágico agudo, mas também como um potente indutor de estado pró-trombótico. Pacientes com fraturas do anel pélvico ou do acetábulo apresentam risco substancialmente aumentado de tromboembolismo venoso (TEV), incluindo trombose venosa profunda (TVP) e embolia pulmonar^2^. A fisiopatologia desse processo é multifatorial e reflete, de maneira clássica, a tríade de Virchow: dano endotelial causado pelo trauma e por procedimentos cirúrgicos; estase decorrente da imobilização prolongada e da compressão extrínseca das veias; e estado de hipercoagulabilidade resultante da resposta inflamatória sistêmica e da ativação da cascata hemostática no contexto do politrauma^3^. A combinação desses mecanismos cria um ambiente trombogênico persistente, que pode se estender por semanas após o insulto inicial.

As diretrizes internacionais recomendam a introdução precoce da profilaxia farmacológica para TEV em pacientes traumatizados, tão logo o risco hemorrágico esteja adequadamente controlado^4^. Contudo, no contexto específico do trauma pélvico grave, essa decisão frequentemente envolve um dilema clínico relevante. Em indivíduos com sangramento ativo, instabilidade hemodinâmica ou necessidade de intervenções cirúrgicas repetidas, a anticoagulação precoce pode, teoricamente, agravar as perdas sanguíneas ou comprometer as estratégias de controle hemorrágico^5^. Assim, o equilíbrio entre os riscos trombótico e hemorrágico assume papel central na tomada de decisão, especialmente em centros de trauma de alta complexidade.

Na prática assistencial, observa-se que pacientes com maior gravidade fisiológica tendem a receber tromboprofilaxia farmacológica mais tardiamente, após a estabilização hemodinâmica e o controle definitivo ou temporário do sangramento. Embora tal conduta seja compreensível sob o prisma hemostático, persiste a preocupação de que o atraso no início da anticoagulação possa aumentar o risco de eventos trombóticos, particularmente em indivíduos já expostos a intenso estímulo inflamatório e transfusional. Estudos observacionais prévios apresentam resultados heterogêneos, frequentemente limitados pela inclusão de populações não específicas de pacientes traumatizados e pela ausência de ajuste adequado para marcadores objetivos de gravidade clínica^6^.

Do ponto de vista da cirurgia vascular e da medicina vascular hospitalar, é fundamental compreender se o momento de início da tromboprofilaxia exerce impacto independente sobre a incidência de TVP em pacientes com trauma pélvico ou se o risco tromboembólico é predominantemente determinado pela magnitude da resposta fisiológica sistêmica ao trauma. A identificação de determinantes independentes de TVP nesse contexto pode contribuir para o desenvolvimento de estratégias de profilaxia mais individualizadas, com melhor equilíbrio entre segurança hemorrágica e proteção antitrombótica.

Diante desse cenário, o presente estudo teve como objetivo avaliar, em uma população específica de pacientes com trauma pélvico atendidos em um centro de trauma de nível I, se o início tardio da profilaxia farmacológica está associado a uma maior incidência de TVP em comparação com o início precoce, após ajuste para variáveis relacionadas à gravidade clínica. A hipótese testada foi a de que, uma vez considerados marcadores objetivos de instabilidade e gravidade hemorrágica, o início tardio da anticoagulação não estaria independentemente associado ao aumento da incidência de TVP durante a internação hospitalar.

## MÉTODOS

Trata-se de um estudo observacional de coorte retrospectiva, conduzido em um hospital terciário de alta complexidade, classificado como centro de trauma de nível I, o Hospital de Base de São José do Rio Preto, Brasil. O período de inclusão compreendeu de janeiro de 2022 a dezembro de 2023. O estudo foi aprovado pelo Comitê de Ética em Pesquisa da instituição (CAAE: 67737823.0.0000.5415, número do parecer 6.050.468).

A identificação dos pacientes foi realizada por meio de busca sistemática no banco de dados institucional do serviço de trauma, com base em registros eletrônicos hospitalares, prontuários físicos e no sistema de codificação diagnóstica. Inicialmente, foram identificados todos os pacientes adultos admitidos por trauma no período do estudo. Em seguida, aplicou-se um filtro específico para identificar aqueles com diagnóstico de lesão pélvica confirmado por tomografia computadorizada realizada na admissão hospitalar. A definição de trauma pélvico incluiu fraturas do anel pélvico, fraturas acetabulares e lesões complexas associadas, independentemente da necessidade de tratamento cirúrgico.

Após essa triagem inicial, foram aplicados critérios de elegibilidade previamente definidos. Foram incluídos pacientes com idade igual ou superior a 18 anos, confirmação tomográfica de lesão pélvica e permanência hospitalar suficiente para a avaliação do desfecho trombótico. Foram excluídos os pacientes que evoluíram para óbito nas primeiras 48 horas de internação, considerando-se que, nesse intervalo, não houve tempo clínico suficiente para a instituição sistemática da profilaxia farmacológica e o monitoramento do desfecho. Também foram excluídos os pacientes com contraindicação absoluta à anticoagulação durante toda a internação, como sangramento intracraniano não estabilizado ou coagulopatia grave persistente, bem como aqueles que não realizaram ultrassonografia Doppler dos membros inferiores durante a internação, o que impossibilitou a avaliação objetiva do desfecho primário. Após a aplicação sequencial desses critérios, obteve-se uma amostra final de 62 pacientes elegíveis para análise. Esse processo seguiu as recomendações da checklist STROBE para a descrição transparente do fluxo de participantes.

A coleta de dados foi realizada por meio de revisão detalhada dos prontuários eletrônicos e físicos, contemplando variáveis demográficas, mecanismo do trauma, parâmetros clínicos na admissão, necessidade de intervenções cirúrgicas, uso de hemocomponentes, tempo de internação, tipo e momento de início da profilaxia farmacológica e ocorrência de TVP. Foram registrados indicadores de gravidade fisiológica, incluindo o índice de choque (*Shock Index* [SI]) e a ativação do protocolo de transfusão maciça, além da presença de sinais clínicos de choque na admissão.

A exposição principal foi o momento de início da profilaxia farmacológica para TVP, categorizado em dois grupos: início precoce, definido como a administração da primeira dose de anticoagulante nas primeiras 48 horas após a admissão hospitalar; e início tardio, definido como a administração após esse período. O ponto de corte de 48 horas foi estabelecido com base em práticas clínicas institucionais consolidadas e em evidências previamente descritas na literatura especializada. A decisão quanto ao momento de início da anticoagulação foi individualizada e realizada pela equipe assistente, considerando a estabilidade hemodinâmica, a necessidade de transfusão sanguínea, a presença de sangramento ativo, os achados dos exames de imagem, a necessidade de intervenções cirúrgicas ou endovasculares e a avaliação global do risco hemorrágico.

O desfecho primário foi a ocorrência de TVP diagnosticada por ultrassonografia Doppler dos membros inferiores durante a internação hospitalar. A TVP foi considerada presente quando identificada trombose no sistema venoso profundo, independentemente da presença de manifestações clínicas. A ultrassonografia Doppler dos membros inferiores seguia protocolo institucional e era realizada sistematicamente em pacientes internados em unidade de terapia intensiva (UTIs) a partir do quinto dia de internação ou diante de suspeita clínica de TVP. Nos pacientes internados em enfermaria, o exame era solicitado conforme critérios assistenciais padronizados da equipe de trauma, considerando-se os fatores de risco tromboembólico e a evolução clínica. Todos os pacientes incluídos na amostra realizaram pelo menos uma ultrassonografia Doppler dos membros inferiores durante a internação hospitalar, independentemente da presença de sintomas. Dessa forma, a avaliação do desfecho trombótico seguiu protocolo assistencial institucional previamente estabelecido, garantindo que todos os pacientes fossem submetidos a pelo menos uma avaliação objetiva para TVP durante a internação.

Embora o delineamento retrospectivo limite as inferências causais, a análise ajustada buscou mitigar potenciais fatores de confusão clinicamente relevantes, conferindo maior robustez à interpretação dos achados.

A análise estatística foi conduzida em etapas sequenciais. Inicialmente, realizou-se uma análise descritiva das variáveis, com o cálculo de medidas de tendência central e de dispersão para variáveis quantitativas e de frequências absolutas e relativas para as variáveis categóricas. As variáveis categóricas foram comparadas por meio do teste do qui-quadrado ou do teste exato de Fisher, quando apropriado. A normalidade dos dados quantitativos foi avaliada pelo teste de Kolmogorov-Smirnov. Considerando a distribuição não normal da maioria das variáveis contínuas, utilizou-se o teste de Mann-Whitney para comparações entre grupos independentes. As análises de correlação foram realizadas por meio do coeficiente de correlação de Spearman, com classificação da magnitude das correlações conforme valores previamente definidos. O nível de significância adotado foi de p ≤ 0,05.

Para avaliar a associação entre o momento de início da tromboprofilaxia farmacológica e a ocorrência de TVP, realizou-se inicialmente uma análise univariada. Em seguida, foi construído um modelo de regressão logística multivariada, tendo como variável dependente a ocorrência de TVP (sim/não) e como principal variável independente o momento de início da anticoagulação (precoce *versus* tardia). Considerando o número limitado de eventos observados, o modelo multivariado final foi restrito a três variáveis, selecionadas com base na plausibilidade clínica e na relevância como potenciais fatores de confusão, a fim de reduzir o risco de superajuste e preservar a estabilidade das estimativas. Assim, além da principal variável de exposição, foram incluídas a idade, como variável contínua, e a ativação do protocolo de transfusão maciça, como marcador de gravidade fisiológica. As estimativas foram expressas como *odds ratio* (OR), com os respectivos intervalos de confiança de 95% (IC95%). Não houve dados ausentes para as principais variáveis incluídas nas análises.

Por se tratar de um estudo retrospectivo baseado em uma amostra de conveniência, foram incluídos todos os pacientes elegíveis identificados no período previamente definido, após a aplicação dos critérios de inclusão e exclusão. Dessa forma, não foi realizado cálculo amostral prévio para determinar o número de participantes, uma vez que o tamanho da amostra foi condicionado à disponibilidade de casos no banco de dados institucional durante o período analisado. Ainda assim, foi realizada uma estimativa complementar do tamanho amostral necessário para a comparação entre duas proporções independentes, com finalidade interpretativa. Para esse cálculo, foram consideradas as proporções de TVP observadas nos grupos de início precoce e tardio da tromboprofilaxia farmacológica, adotando-se um nível de significância de 5% e poder estatístico de 80%, parâmetros convencionalmente utilizados em cálculos amostrais para estudos clínicos. Essa análise foi utilizada para contextualizar a precisão das estimativas e a capacidade do estudo de detectar diferenças entre os grupos avaliados.

## RESULTADOS

Foram incluídos 62 pacientes com diagnóstico de trauma pélvico contuso, com idade média de 47,71±19,91 anos, evidenciando ampla variabilidade etária na coorte. Observou-se predominância do sexo masculino (66,1%), e o mecanismo de trauma foi classificado como de alta energia em 80,6% dos casos, caracterizando uma população exposta a elevado grau de transferência de energia. No atendimento pré-hospitalar, 21% dos pacientes apresentavam sinais clínicos de choque. A reposição volêmica com cristaloides foi realizada em 38,7% da amostra, com administração de volume ≤ 1.000 mL em 25,8% e > 1.000 mL em 12,9%. O ácido tranexâmico foi administrado ainda no ambiente pré-hospitalar em 17,7% dos casos.

Na admissão hospitalar, 8,1% dos pacientes mantinham sinais clínicos de hipovolemia e 11,3% apresentavam sangramento ativo evidente. O protocolo de transfusão maciça foi ativado em 8,1% da amostra. O SI apresentou média de 0,82±0,37 e mediana de 0,76, evidenciando variabilidade na resposta hemodinâmica inicial. O escore ABC apresentou valores predominantemente baixos: 74,2% dos pacientes apresentando escore 0; 17,7%, escore 1; 6,5%, escore 2; e 1,6%, escore 3.

Fraturas associadas em outras regiões anatômicas foram identificadas em 59,7% dos pacientes, indicando alta frequência de politrauma. A cirurgia de controle de danos foi necessária em 14,5% da amostra. A correção cirúrgica da fratura pélvica foi realizada em 58,1% dos casos, enquanto 40,3% foram submetidos a correção de fraturas associadas. O tempo médio de permanência em UTI foi de 12,98±14,46 dias, com mediana de 7 dias, enquanto a permanência em enfermaria apresentou média de 15,08±17,10 dias e mediana de 9 dias, evidenciando grande dispersão na evolução clínica hospitalar (Tabela 1).

**Tabela 1:** Características demográficas e clínicas iniciais (n = 62).

Variável	Valor
Idade (anos)	47,71±19,91
Sexo masculino	66,1%
Trauma de alta energia	80,6%
Choque pré-hospitalar	21%
Reposição com cristaloides	38,7%
≤ 1.000 mL	25,8%
> 1.000 mL	12,9%
Ácido tranexâmico pré-hospitalar	17,7%
Hipovolemia na admissão	8,1%
Sangramento ativo evidente	11,3%
Protocolo de transfusão maciça	8,1%
SI (média ± DP)	0,82±0,37
SI (mediana)	0,76
Escore ABC 0	74,2%
Escore ABC 1	17,7%
Escore ABC 2	6,5%
Escore ABC 3	1,6%
Fraturas associadas	59,7%
Cirurgia de controle de danos	14,5%
Cirurgia para fratura pélvica	58,1%
Cirurgia para outras fraturas	40,3%
UTI (dias, média ± DP)	12,98±14,46
UTI (mediana)	7
Enfermaria (dias, média ± DP)	15,08±17,10
Enfermaria (mediana)	9

DP = desvio-padrão; SI = índice de choque (*Shock Index*); UTI = unidade de terapia intensiva.

Todos os pacientes receberam profilaxia farmacológica para tromboembolismo venoso durante a internação. A enoxaparina foi utilizada em 54,8% dos casos, enquanto 45,2% receberam heparina não fracionada. O início da profilaxia ocorreu em até 48 horas após a admissão hospitalar em 45,2% dos pacientes, ao passo que, em 54,8%, ocorreu após 48 horas. O tempo médio até o início da tromboprofilaxia farmacológica foi de 3,44±2,15 dias. A comparação detalhada das características basais entre os grupos de início precoce (≤ 48 horas) e tardio (> 48 horas) da profilaxia farmacológica é apresentada na Tabela 2. Não foram observadas diferenças estatisticamente significativas entre os grupos quanto à idade, ao SI, à presença de choque na admissão ou à necessidade de cirurgia de controle de danos, embora o grupo de início tardio tenha apresentado maior proporção de ativação do protocolo de transfusão maciça. Essa análise descritiva foi realizada para avaliar a possibilidade de confusão por indicação.

**Tabela 2:** Comparação entre início precoce (≤ 48 h) e tardio (> 48 h).

Variável	≤ 48 h (n = 28)	> 48 h (n = 34)	Valor de p
Idade (média ± DP)	46,36±18,52	48,85±21,10	0,642
Índice de choque (*Shock Index*) (média ± DP)	0,79±0,31	0,85±0,41	0,588
Choque na admissão (%)	7,1%	8,8%	0,812
Protocolo de transfusão maciça (%)	3,6%	11,8%	0,284
Cirurgia de controle de danos (%)	10,7%	17,6%	0,482

DP = desvio-padrão; h = horas.

A TVP foi confirmada por ultrassonografia Doppler em 32,3% dos pacientes (n = 20). O tempo médio até o diagnóstico foi de 19,17±14,26 dias após o trauma, com mediana de 13,5 dias e variação de 6 a 55 dias. Entre os casos confirmados, 50% dos pacientes apresentavam edema no momento do diagnóstico, 10% relataram dor e 40% eram assintomáticos, com diagnóstico estabelecido por meio de rastreamento ultrassonográfico (Tabela 3).

**Tabela 3:** Profilaxia anticoagulante e incidência de TVP.

Variável	Grupo	Valor
Anticoagulante	Enoxaparina	54,8%
Anticoagulante	Heparina não fracionada	45,2%
Início da profilaxia	≤ 48 h	45,2%
Início da profilaxia	> 48 h	54,8%
Tempo até o início (dias)	Média ± DP	3,44±2,15
Incidência de TVP	Global	32,3% (n = 20)
TVP – Início ≤ 48 h	-	28,6% (8/28)
TVP – Início > 48 h	-	35,3% (12/34)
Tempo até TVP (dias)	Média ± DP	19,17±14,26
Tempo até TVP (dias)	Mediana (intervalo)	13,5 (6-55)
TVP sintomática	Edema	50%
TVP sintomática	Dor	10%
TVP assintomática	-	40%

DP = desvio-padrão; h = horas; TVP = trombose venosa profunda.

Na análise bivariada, observou-se que 12 dos 34 pacientes (35,3%) que iniciaram a tromboprofilaxia farmacológica após 48 horas desenvolveram TVP, enquanto oito dos 28 pacientes (28,6%) que a iniciaram em até 48 horas apresentaram o evento, correspondendo a uma diferença absoluta de 6,7 pontos percentuais, sem significância estatística (p = 0,598). A gravidade do trauma pélvico, estimada pelo número de fraturas identificadas, não apresentou associação estatisticamente significativa com a ocorrência de TVP (p = 0,7525).

Foi ajustado um modelo de regressão logística multivariada considerando a ocorrência de TVP como variável dependente. O modelo final foi definido previamente com base na plausibilidade clínica e na limitação imposta pelo número de eventos observados, a fim de reduzir o risco de superajuste e preservar a estabilidade das estimativas. Assim, foram incluídas três variáveis no modelo ajustado: início precoce da tromboprofilaxia (≤ 48 horas), como principal variável independente de interesse; idade, considerada uma variável contínua com potencial influência biológica sobre risco trombótico; e ativação do protocolo de transfusão maciça, utilizada como marcador objetivo de gravidade fisiológica e instabilidade hemorrágica inicial.

No modelo multivariado ajustado, o início precoce da tromboprofilaxia farmacológica apresentou OR de 0,65 (IC95% 0,21-2,00; p = 0,456). O amplo intervalo IC observado indica imprecisão da estimativa, compatível com o número limitado de eventos na coorte. A idade apresentou OR de 1,01 por ano adicional (IC95% 0,98-1,04; p = 0,452). A ativação do protocolo de transfusão maciça apresentou OR de 16,09 (IC95% 0,89-292,13; p = 0,061), também com amplo IC, de modo que a magnitude dessa estimativa deve ser interpretada com cautela (Tabela 4).

**Tabela 4:** Regressão logística multivariada para ocorrência de trombose venosa profunda.

Variável	OR ajustado	IC95%	Valor de p
Anticoagulação precoce (≤ 48 h)	0,65	0,21-2,00	0,456
Protocolo de transfusão maciça	16,09	0,89-292,13	0,061
Idade (por ano adicional)	1,01	0,98-1,04	0,452

IC95% = intervalo de confiança de 95%; h = horas; OR = *odds ratio*.

A estimativa pontual inferior a 1 para o início precoce da tromboprofilaxia farmacológica manteve-se após o ajuste para idade e gravidade hemorrágica, sem evidência de associação independente com a ocorrência de TVP na coorte analisada. A ativação do protocolo de transfusão maciça apresentou a maior magnitude de associação no modelo, reforçando a relevância da gravidade fisiológica inicial na estratificação do risco trombótico em pacientes com trauma pélvico. Esses achados sugerem que o perfil sistêmico de instabilidade hemorrágica pode exercer maior influência sobre o risco de TVP do que o intervalo até o início da tromboprofilaxia farmacológica.

## DISCUSSÃO

A TVP no contexto do trauma pélvico permanece um desafio clínico de grande relevância, mesmo em centros de trauma estruturados e sob uso sistemático de profilaxia farmacológica. A literatura demonstra que as fraturas do anel pélvico e acetabulares estão entre as lesões ortopédicas com maior risco de tromboembolismo venoso, particularmente quando associadas a politrauma, necessidade de internação em UTI e múltiplas intervenções cirúrgicas^1,7,8^. Assim, a incidência observada nesta coorte não deve ser interpretada como um evento isolado, mas como uma manifestação esperada de um fenótipo biológico altamente trombogênico.

O achado central deste estudo — a ausência de associação independente entre o início tardio da tromboprofilaxia farmacológica e a ocorrência de TVP após ajuste para idade e ativação do protocolo de transfusão maciça — merece análise cuidadosa. Diretrizes europeias e internacionais recomendam o início precoce da profilaxia farmacológica assim que o risco hemorrágico estiver controlado^4^; entretanto, reconhecem que pacientes com trauma pélvico grave frequentemente apresentam instabilidade hemodinâmica e necessidade de transfusão, fatores que podem justificar o adiamento prudente da profilaxia^9,10^. Estudos prospectivos recentes sugerem que o início precoce da heparina de baixo peso molecular é seguro em pacientes com fraturas pélvicas estabilizadas^11,12^, porém, esses trabalhos, em geral, incluem populações com critérios rigorosos de estabilização e menor proporção de pacientes submetidos a transfusão maciça.

O número limitado de eventos observados restringe o poder estatístico do estudo para detectar diferenças modestas entre os grupos. O amplo IC da principal variável de exposição sugere possibilidade de erro tipo II, não sendo possível excluir a existência de uma associação de pequena ou moderada magnitude entre o momento de início da tromboprofilaxia farmacológica e a ocorrência de TVP.

Embora o cálculo amostral prévio não fosse obrigatório para o delineamento retrospectivo adotado, baseado na inclusão de todos os pacientes elegíveis durante o período analisado, a estimativa complementar do tamanho amostral necessário para detectar uma diferença da magnitude observada contribui para contextualizar os resultados do estudo. Considerando as proporções observadas de TVP nos grupos de início tardio e precoce da tromboprofilaxia farmacológica, respectivamente 28,6% e 35,3%, correspondendo a uma diferença absoluta de 6,7 pontos percentuais, com nível de significância de 5% e poder estatístico de 80%, seria necessária uma amostra aproximada de 759 pacientes por grupo, totalizando cerca de 1.518 pacientes em alocação 1:1, para detectar uma diferença dessa magnitude entre os grupos. Dessa forma, a ausência de significância estatística observada nesta coorte deve ser interpretada com cautela, pois pode refletir poder estatístico insuficiente para identificar diferenças discretas entre as estratégias avaliadas. Esses achados reforçam que os resultados do presente estudo devem ser interpretados como exploratórios e geradores de hipótese e não como demonstração definitiva de equivalência entre início precoce e tardio da tromboprofilaxia farmacológica.

Ao ajustar o modelo para idade e para a ativação do protocolo de transfusão maciça, utilizada como marcador objetivo de gravidade fisiológica, buscou-se reduzir o potencial de confusão por indicação, frequentemente presente em estudos retrospectivos. Pacientes mais graves tendem a receber a tromboprofilaxia farmacológica mais tardiamente e, simultaneamente, apresentam maior risco trombótico intrínseco. Nesse contexto, os resultados sugerem que fatores relacionados à gravidade sistêmica inicial podem exercer maior influência sobre o risco de TVP do que o momento de início da tromboprofilaxia farmacológica.

Um aspecto particularmente relevante foi a magnitude da associação observada entre a ativação do protocolo de transfusão maciça e a ocorrência de TVP. A literatura demonstra que pacientes submetidos a transfusão maciça apresentam alterações profundas da hemostasia, incluindo coagulopatia inicial seguida por um estado de hipercoagulabilidade reativa^4,13,14^. A resposta inflamatória sistêmica exacerbada, a lesão endotelial difusa e a ativação plaquetária contribuem para um ambiente pró-trombótico persistente^3^. Portanto, os resultados sugerem que o risco tromboembólico no trauma pélvico é multifatorial e pode ser fortemente influenciado pela condição fisiológica global do paciente.

A necessidade de cirurgia para controle de danos merece consideração no contexto clínico do trauma pélvico grave. Esses procedimentos abreviados, frequentemente realizados em pacientes com instabilidade fisiológica, têm como objetivo o controle hemorrágico e estabilização fisiológica^15,16^. Esses pacientes acumulam fatores adicionais de risco pró-trombótico, como imobilização prolongada, múltiplas reoperações e permanência prolongada em UTI. Embora essa variável não tenha sido incluída no modelo multivariado final, ela representa um marcador clínico de gravidade que pode estar associado a maior vulnerabilidade tromboembólica em pacientes com instabilidade fisiológica acentuada.

Outro ponto relevante refere-se ao momento do diagnóstico de TVP, que ocorreu predominantemente em fase tardia da internação. Esse achado é consistente com estudos que demonstram a persistência do risco trombótico ao longo da hospitalização, mesmo após a estabilização clínica inicial^7,8,17^. A hipercoagulabilidade pós-traumática pode persistir por semanas, influenciada pela resposta inflamatória contínua e pelo estímulo cirúrgico repetido^3^. Portanto, a prevenção do tromboembolismo venoso em pacientes com trauma pélvico deve ser compreendida como uma estratégia longitudinal, e não apenas como uma intervenção precoce isolada.

A proporção de eventos assintomáticos detectados por ultrassonografia reforça a relevância do rastreamento por imagem para o diagnóstico de TVP. Estudos que utilizam ultrassonografia Doppler seriada demonstram incidência substancial de TVP subclínica em pacientes com fraturas pélvicas e acetabulares^7,8^. A ausência de um protocolo padronizado de rastreamento pode introduzir viés de detecção, influenciando a incidência observada e dificultando comparações entre diferentes instituições. No entanto, esse cenário reflete a prática clínica de muitos centros e, portanto, confere maior aplicabilidade externa aos achados.

No que diz respeito ao tipo de anticoagulante, metanálises indicam que a heparina de baixo peso molecular pode apresentar discreta vantagem na prevenção do tromboembolismo venoso em pacientes traumatizados^18^. Entretanto, fatores institucionais, ajustes de dose e características individuais dos pacientes podem atenuar as diferenças entre os agentes. A ausência de evidência de associação independente entre o tipo de heparina e a ocorrência de TVP nesta análise sugere que, no contexto estudado, a gravidade do trauma e o estado fisiológico do paciente podem exercer influência mais relevante do que a escolha específica do anticoagulante.

Metodologicamente, o delineamento retrospectivo impõe limitações, incluindo a possibilidade de confusão residual decorrente de variáveis não mensuradas, como parâmetros laboratoriais dinâmicos de coagulação, grau de imobilização e variações na estratégia de reabilitação precoce. Além disso, o número limitado de eventos restringiu o número de variáveis que puderam ser incluídas no modelo multivariado final, aumentando a possibilidade de confusão residual. A exclusão de pacientes que evoluíram para óbito nas primeiras 48 horas foi necessária para a adequada avaliação da exposição, mas pode ter introduzido viés de sobrevivência.

No contexto nacional, as recomendações da Sociedade Brasileira de Angiologia e de Cirurgia Vascular sobre tromboembolismo venoso^19^ reforçam a importância da estratificação individualizada dos riscos trombótico e hemorrágico em pacientes hospitalizados, especialmente naqueles submetidos a trauma, cirurgia, imobilização prolongada ou internação em UTI. Essa perspectiva é particularmente relevante no trauma pélvico grave, no qual coexistem fatores fortemente pró-trombóticos, como lesão endotelial, resposta inflamatória sistêmica, estase venosa, politrauma, necessidade de procedimentos cirúrgicos e permanência prolongada no leito, ao mesmo tempo em que o risco hemorrágico inicial pode limitar ou postergar o início seguro da tromboprofilaxia farmacológica. Dessa forma, os achados do presente estudo devem ser interpretados à luz desse equilíbrio clínico. Embora a tromboprofilaxia farmacológica deva ser instituída precocemente sempre que houver segurança hemostática, seu início mais tardio em pacientes com instabilidade fisiológica pode refletir maior gravidade basal, e não necessariamente representar um fator independente de risco para TVP. Essa interpretação está em consonância com a necessidade, enfatizada pelas diretrizes nacionais, de individualizar a prevenção da TVP de acordo com o perfil clínico, o risco de sangramento e a evolução hemodinâmica de cada paciente.

Apesar dessas limitações, o estudo contribui ao avaliar uma população específica de pacientes com trauma pélvico em um centro de trauma nível I, com ajuste para um marcador objetivo de gravidade fisiológica. Os achados sugerem que o risco tromboembólico é fortemente influenciado pela condição fisiológica global do paciente^4,10,20^. Nesta coorte, o início mais tardio da profilaxia farmacológica, quando clinicamente justificado por instabilidade hemodinâmica ou risco hemorrágico, não apresentou associação independente com a ocorrência de TVP após ajuste para idade e gravidade hemorrágica. Esses resultados devem ser interpretados à luz das limitações metodológicas descritas e reforçam a importância de uma avaliação individualizada do equilíbrio entre os riscos hemorrágico e trombótico em pacientes com trauma pélvico grave.

## CONCLUSÃO

Em pacientes com trauma pélvico contuso atendidos em um centro de trauma de nível I, não foi possível demonstrar associação independente entre início da tromboprofilaxia farmacológica após 48 horas e maior ocorrência de TVP após ajuste para idade e gravidade hemorrágica. Considerando o número limitado de eventos e a amplitude dos intervalos de confiança, estudos com maior poder estatístico são necessários para confirmar esses achados e definir com maior precisão o impacto do momento de início da tromboprofilaxia farmacológica nesse cenário clínico.

## Data Availability

The data that support this study are available on request from the corresponding author, JMES, because sharing these data is subject to ethical restrictions and participants’ confidentiality must be protected.
